# An Innovative Drug Repurposing Approach to Restrain Endometrial Cancer Metastatization

**DOI:** 10.3390/cells12050794

**Published:** 2023-03-03

**Authors:** Federica Torricelli, Elisabetta Sauta, Veronica Manicardi, Vincenzo Dario Mandato, Andrea Palicelli, Alessia Ciarrocchi, Gloria Manzotti

**Affiliations:** 1 Laboratory of Translational Research, Azienda USL-IRCCS di Reggio Emilia, Viale Risorgimento 80, 42123 Reggio Emilia, Italy; 2 Department of Electrical, Computer and Biomedical Engineering, University of Pavia, 27100 Pavia, Italy; 3 Clinical and Experimental Medicine PhD Program, University of Modena and Reggio Emilia, 41125 Modena, Italy; 4 Unit of Obstetrics and Gynaecology, Azienda USL-IRCCS di Reggio Emilia, 42123 Reggio Emilia, Italy; 5 Pathology Unit, Department of Oncology and Advanced Technologies, Azienda USL-IRCCS di Reggio Emilia, 42123 Reggio Emilia, Italy

**Keywords:** drug repurposing, endometrial cancer, gene expression, metastasis, PI3K pathway

## Abstract

Background: Endometrial cancer (EC) is the most common gynecologic tumor and the world’s fourth most common cancer in women. Most patients respond to first-line treatments and have a low risk of recurrence, but refractory patients, and those with metastatic cancer at diagnosis, remain with no treatment options. Drug repurposing aims to discover new clinical indications for existing drugs with known safety profiles. It provides ready-to-use new therapeutic options for highly aggressive tumors for which standard protocols are ineffective, such as high-risk EC. Methods: Here, we aimed at defining new therapeutic opportunities for high-risk EC using an innovative and integrated computational drug repurposing approach. Results: We compared gene-expression profiles, from publicly available databases, of metastatic and non-metastatic EC patients being metastatization the most severe feature of EC aggressiveness. A comprehensive analysis of transcriptomic data through a two-arm approach was applied to obtain a robust prediction of drug candidates. Conclusions: Some of the identified therapeutic agents are already successfully used in clinical practice to treat other types of tumors. This highlights the potential to repurpose them for EC and, therefore, the reliability of the proposed approach.

## 1. Introduction

Endometrial cancer (EC) is the most frequent gynecologic tumor [1]. Its incidence and associated mortality are increasing over the past years. The gold standard treatment for EC at an early stage is total hysterectomy with bilateral adnexectomy and sentinel lymph node biopsy or systematic pelvic/lumbar-aortic lymphadenectomy in case of increased risk of lymph node metastases. In advanced EC, chemotherapy is indicated and should precede debulking surgery if complete cytoreduction is achievable. Thus, a major limitation of a successful first-line treatment is the presence of metastasis. Despite many patients having a good prognosis, advanced or recurrent EC have a poor prognosis, and metastatic or recurrent tumors are partially or not responsive at all [2]. It emerges as an urgent need to identify new druggable targets to circumvent the tumor resistance mechanism and to target specifically metastatic tumors.

In recent years, thanks to the acquired deep knowledge about genetic and molecular assets of many types of cancer, a growing number of targeted therapies have become available but, at present, few are approved for EC [3,4]. Drug repurposing is the application of old drugs to new indications. Repositioning already-approved drugs presents obvious advantages. Knowing the safety, toxicity, pharmacokinetic, pharmacodynamic, and metabolic properties of a compound significantly reduces risk, costs, and the time required to register the indication, as compared to a new chemical entity [5,6,7]. This approach could potentially provide new ready-to-use therapeutic options for aggressive and resistant cancers and has already been largely employed in different tumor settings [8,9,10,11,12,13,14,15,16,17]. Many different computational methodologies have been developed for this purpose, mainly data-driven [18]. One of the most used is based on the differential gene expression signature (dGES) and compares disease or clinical phenotypes against a reference condition [19,20]. The obtained signature could be used as input for various methods to make drug-disease associations, returning a list of compounds that could potentially revert the matched signature and the disease phenotype itself. Another increasingly used methodology is the pathway-based analysis which can help to identify the most deregulated processes, allowing to prioritize drug targets within the drug-gene association procedure [21,22,23].

In this work, we introduce a new robust integrated repurposing approach identifying new ready-to-use therapeutic options for metastatic EC.

## 2. Materials and Methods

### 2.1. Computational Framework Overview

**Figure 1:**
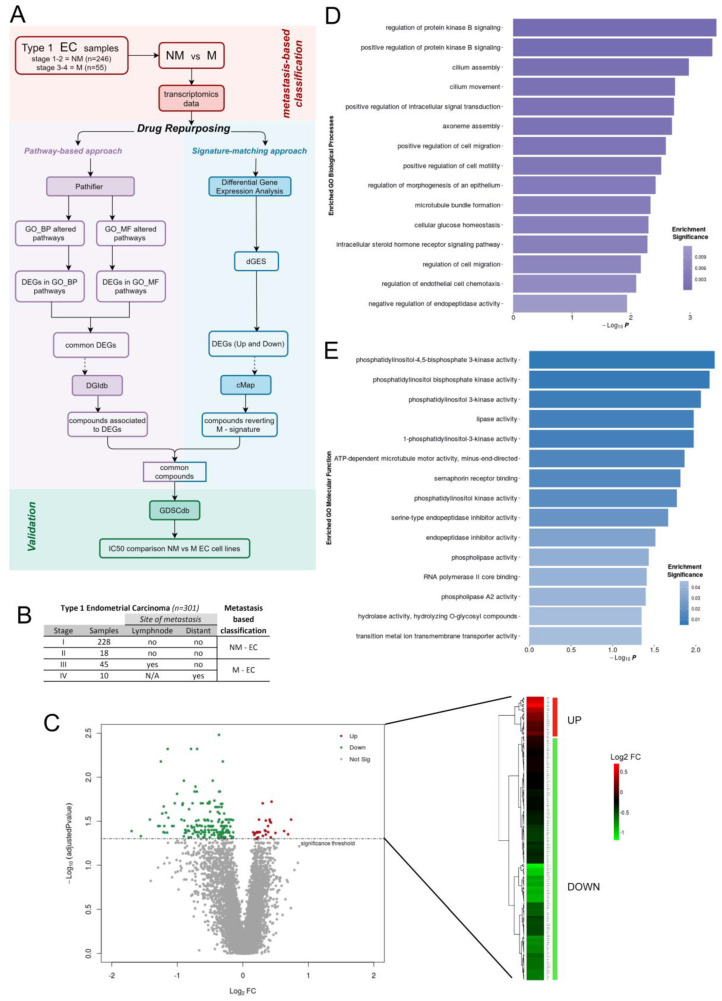
Comparison of metastatic and non-metastatic EC transcriptomic profiles: (**A**) Computational framework overview of the integrative drug repurposing pipeline. (**B**) Table summarizing metastasis-based re-classification of Type 1 EC samples. (**C**) Volcano plot and a heat map showing significantly UP and DOWN-regulated genes constituting dGES (FDR < 0.05). (**D**,**E**) Functional enrichment analysis of dGES, Gene Ontology-Biological Processes (GO_BO), and Gene Ontology-Molecular Functions, respectively.

The drug repurposing integrative pipeline used in the present work is shown in Figure 1A. Briefly, we retrieved publicly available transcriptomics data of endometrial cancer samples and we stratified patients according to the FIGO Stage, classifying them as non-metastatic (NM) or metastatic (M). With the final aim to discover promising drug candidates, we compared these two groups with an integrated approach using two computational strategies, one signature-matching and one pathway-based, obtaining, respectively, a differential Gene Expression Signature (dGES) and a ranked list of the most deregulated pathways underlying metastatization. All Differential Expressed Genes (DEGs) participating in these pathways were identified as the most perturbed. Next, the dGES was used to query the Connectivity Map (cMap) database, while the perturbed genes associated with deregulated pathways were used to query the Drug-Gene Interaction database (DGIdb) [24], for identifying potential drug-gene pairs. Finally, the obtained drug lists were merged, and the remaining compounds were in-silico validated through the Genomics of Drug Sensitivity in Cancer (GDSC) database.

### 2.2. Transcriptomics Data Collection and Processing

Transcriptomics data used in this work belong to the Uterine Corpus Endometrial Carcinoma (UCEC) project of The Cancer Genome Atlas (TCGA) and were downloaded by the R package “UCSCXenaTools” [25]. Since our main purpose was to compare NM and M EC, we based on the FIGO (International Federation of Gynecology and Obstetrics) classification which divides EC into four stages, with an increasing level of aggressiveness and dissemination, based on histologic differentiation and intra-operative evaluation of abdominal and pelvic spreading [26,27,28]. Only cases described in the study by Cancer Genome Atlas Research Network, Kandoth C, Schultz N, et al. [29] were selected, since in this work Stages annotation uniformly referred to the FIGO stage nomenclature of 2009, without misinterpretations (as specified in the “Supplementary Method S1” of the cited work [29]). Moreover, we considered only Type 1 EC, which is the most common histotype accounting for 80–90% of total cases and includes only endometrioid tumors [30]. We also excluded cases with “unknown” histotypes. To properly define a sample as metastatic or not, we considered clinical and histopathological features and used, as a reference, the FIGO stage, considering both Stage 4 and Stage 3 as metastatic diseases (metastatic—M). Conversely, we grouped Stages 1 and 2 which never present metastasis (non-metastatic—NM) (Figure 1B). Thus, we obtained a final dataset composed of 246 samples for the NM group, and of 55 samples for the M one, for which RNA-sequencing, clinical, and histopathological data were available. The specimens used in this database were primary tumors, collected at diagnosis and only from patients with no prior treatment before surgery.

All data were downloaded from TCGA, which is open to the public under certain restrictions, therefore no ethical approval was needed.

### 2.3. Differential Gene Expression Analyses

Differential gene expression analysis was performed in the R environment (v3.6.3) (R Foundation for Statistical, Vienna, Austria) comparing the mean of the expression of each gene in M and NM groups by Kruskal Wallis statistical test and calculating the false discovery rate (FDR) using the Benjamini-Hochberg correction for multiple hypothesis testing to identify statistically significant deregulated genes (FDR < 0.05). For the definition of a consistent dGES, only protein-coding genes with a minimum mean expression higher than 0.5 log2 (FPKM + 1) in both groups were selected. The same statistical analysis was performed comparing Stage I vs. Stage II and Stage III vs. Stage IV.

### 2.4. Functional Enrichment Analysis

Enrichment pathways analyses were performed exploiting Gene Ontologies Biological Process (GO_BP) and Molecular Function (GO_MF) as references using the EnrichR R package [31]. Pathways were considered significantly enriched with a significance threshold of 0.05 (FDR < 0.05).

### 2.5. Drug Repurposing Integrated Approach

#### 2.5.1. Signature-Matching Approach

The obtained dGES was used as input of the cMap (v1.1.1.43, dataset v1.1.1.2, accessed via https://clue.io) (accessed on 9 November 2020) [32,33]. cMap returns as output a list of perturbagenes ranked accordingly to a connectivity score which incorporates a *p*-value corrected for multiple hypothesis testing using the false discovery rate method [33,34]. Since none of the cell lines present on the cMap database at the time of our query were of endometrial cancer, we used the option “summary” which, given a set of connectivity scores for a particular perturbagen, summarizes those scores across all the cell lines tested. cMap output comprises four perturbagene types: gene over-expression and gene-knockdown (which are not considered in this study), compounds (which are our main interest), and Perturbagen Class (PCL) of compounds based on their strong connectivity to each other and shared mechanisms of action (MoA). We evaluated all compounds and PCL with a negative connectivity score, thus applying the reversal method [35,36,37,38,39], and we used a threshold of −60 to have a broader perspective of the molecules with potential reverse connection to our dGES.

#### 2.5.2. Pathway-Based Approach

We applied the Pathifier algorithm [40] using the default parameters setting on our stratified expression dataset, comparing M and NM patients’ profiles. As prior information, we considered GO_BP (number of pathways = 5103) and GO_MF (number of pathways = 1151). Integrating these gene assignments to known pathways with expression data, Pathifier independently quantifies, for a given pathway and each sample, a pathway deregulation score (PDS), representing the deviation of a sample from the reference condition (NM samples). Graphically, this is represented by a cloud of points (each of which is a sample) whose variation is recapitulated by the calculation of a “principal curve”. In this dimensional space, PDS is the distance along the curve between a sample and a reference point, defined as the centroid of a reference set of samples (NM samples). Analyzed pathways were clustered using the assigned PDS obtained from the two Pathifier analyses using SPIN (Sorting Points Into Neighborhoods), an unsupervised sorting method [41] able to capture the gradual changes underlying heterogeneous expression data as a distance matrix to the cluster pathways that share a similar deregulation profile. The resulting matrixes were graphically represented as similarity heatmap using Matplotlib [42] within a Python environment. We then ranked all pathways using the median (PDSM) estimated on the PDS of M samples, assigning, to each pathway, a unique PDSM value. To further filter the obtained pathways, we then considered only those with PDSM > 0.6 and those that have, among interacting genes, differentially expressed genes derived from the dGES. For a graphical purpose, given the high number of perturbed pathways from each of the two Pathifier analyses, we grouped the resulting ranked pathways using a semantic similarity-based approach, which exploited the hierarchical nature of Gene Ontologies. To this aim, we used the Relevance information-content strategy implemented in the rrvgo R package [43], which depends on the frequencies of two GO terms and that of their closest common ancestor in a specific corpus of GO annotations. The resulting similarity matrices were then reduced by applying a threshold of 0.7 on estimated similarity scores. Similar terms were hierarchically clustered using a complete linkage method and the resulting tree was then cut at the corresponding level of 0.7. The obtained list of categories was then manually curated and combined in macro-categories for ease of graphical representation.

The lists of DEGs mapping into the most altered biological processes and molecular functions were then merged for the following drug-target association analysis using the DGIdb database (release 4.2.0, www.dgidb.org) (accessed on 13 July 2021). The DGIdb was queried using Application Programming Interface (API) within the Python environment (v3.5.2, Python Software Foundation, Fredericksburg, VA, USA). Results are returned in JSON format, which was parsed to extract all DEGs associated with one or more drugs and the related type of interaction.

To finally have a unique list of potential drug candidates, the obtained compounds set was then integrated with the cMap output. Since cMap molecules are identified with a Broad Institute (BRD) Identifier, to avoid loss of information during the merging step, we used the PubChem Identifier Exchange Service to convert compound names from DGIdb query in other identifiers. This mapping step was performed using an ad-hoc Python pipeline that returned a final dataset of drugs common to cMap and DGIdb query results.

### 2.6. In silico Validation and Compound Analysis

For each of our previously defined drugs, present in the GDSC database (release 8.3, https://www.cancerrxgene.org/, accessed on 1 July 2021) [24], IC50 results were filtered for “Tissue sub-type: endometrium” and “Disease: endometrial adenocarcinoma”. Since our samples only belonged to Type 1 EC, we excluded all cell lines for which it is not possible to certainly retrieve this information.

The experimental information about the validated compounds were manually curated through the literature mining and querying several databases: DrugBank [44], clinicaltrials.gov, Harmonizome [45], The Drug Repurposing Hub (clue.io/repurposing-app), NCI Drug Dictionary, FDA (https://www.fda.gov/, accessed on 1 July 2021), Inxight Drugs (drugs.ncats.io, accessed on 1 July 2021).

## 3. Results

### 3.1. Differential Gene Expression Profiling Identifies PI3K/AKT/mTOR Pathway as Predominant Feature of Metastatic EC

Differential gene expression analysis between metastatic (M) and non-metastatic (NM) EC identified a robust dGES composed of 212 genes, 29 UP-regulated and 183 DOWN-regulated (Figure 1C, Supplementary Table S1). Functional enrichment analysis revealed that, according to Gene Ontology-Biological Processes (GO_BP), positive regulation of protein kinase B (PKB) signaling is the top-scoring pathway, together with cilium assembly and movement, positive regulation of cell migration and cell mobility processes (Figure 1D). Coherently, among the enriched Gene Ontology-Molecular Functions (GO_MF), the top-scoring pathways mainly concern the phosphatidylinositol kinase activity (Figure 1E). All enriched processes describe a landscape that is connected to endometrial classical features, such as cilium and estrogen-related functions. In addition, a strong correlation between the PI3K/AKT/mTOR pathway and EC metastatization emerged from this analysis. This pathway is strongly associated with EC being altered in more than 93% of patients [29]. However, this is the first time that transcriptional deregulation of this pathway is linked to metastatic progression in EC. The connection between metastasis and the PI3K/AKT/mTOR pathway provided by our data could suggest a new and specific enrolling criterion for clinical trials involving these classes of drugs.

### 3.2. Pathway-Based Analysis Identifies New EC Vulnerability Specifically Related to Metastatization

**Figure 2:**
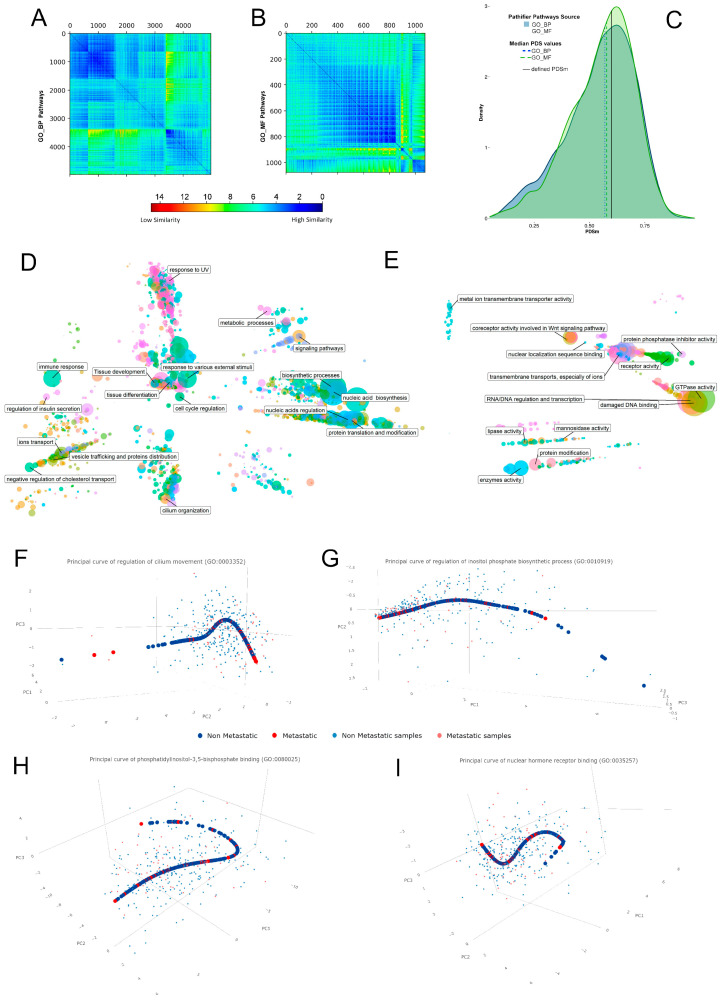
Pathway-based analysis of EC: (**A**,**B**) Heatmaps representing the distribution of the PDS values from GO_BP and GO_MF, respectively. (**C**) The distribution of PDS values identifies the PDSM value. (**D**,**E**) Graphical representation of macro-categories encompassing the pathways found significantly altered by Pathifier, relative to GO_BP and GO_MF respectively. (**F**–**I**) An example of how the cloud of points representing NM and M samples, and the principal curve describing its variation are projected onto the three leading principal components. Four of the most representative pathways found altered are shown.

To deeper investigate the molecular processes underlying metastatization, we expanded the information obtained from the dGES by comparing the transcriptomics profiles of M and NM patients, exploiting the Pathifier algorithm [40]. It translates the gene-level information into pathway-level information, incorporating prior knowledge about functional interactions of biological processes (GO_BP and GO_MF) thus, assigning to each pathway a Pathway Deregulation Score (PDS) which can be clustered based on similarity grade of perturbation (Figure 2A,B). We ranked the resulting deregulations by calculating for each pathway the median PDS on metastatic samples (PDSM) and considered as top scoring those with a PDSM > 0.6 (Figure 2C), thus obtaining a list of significantly deregulated pathways (2096 for GO_BP and 468 for GO_MF). Again, the PI3K/AKT/mTOR pathway was present in these lists, even if the majority of the identified perturbed pathways have never been linked to EC. Pathways from GO_BP vary mainly between nucleic acids regulation and biosynthesis, tissue differentiation and development, response to various external stimuli, vesicle trafficking, proteins and ions distribution, metabolic and biosynthetic processes, and protein translation and modification (Figure 2D). Similarly, the most represented GO_MF are associated with RNA/DNA regulation and transcription, enzyme activity, protein binding, and interaction (receptors activity and complex formation), and transmembrane transports, especially of ions (Figure 2E). Some of these processes are strictly related to cancer progression, while some others are non-obvious, such as “Cilium movement” and “Regulation of inositol phosphate biosynthetic process” (GO_BP), and “Phosphatidylinositol-3,5-bisphosphate binding” and “nuclear hormone receptor binding” (GO_MF) (Figure 2F–I).

These results define a complex molecular landscape of biological alterations leading to EC metastatization and unveil perturbed pathways never considered before as EC vulnerability which can be exploited to target the most aggressive EC.

### 3.3. Drug Repurposing Analysis

Using the results of these analyses, we searched for already approved drugs that could be repurposed on EC by employing two different approaches: (i) querying the cMap to obtain matched drug signatures and, (ii) using the Drug Gene Interaction Database (DGIdb) to derive drug-gene associations.

**Figure 3:**
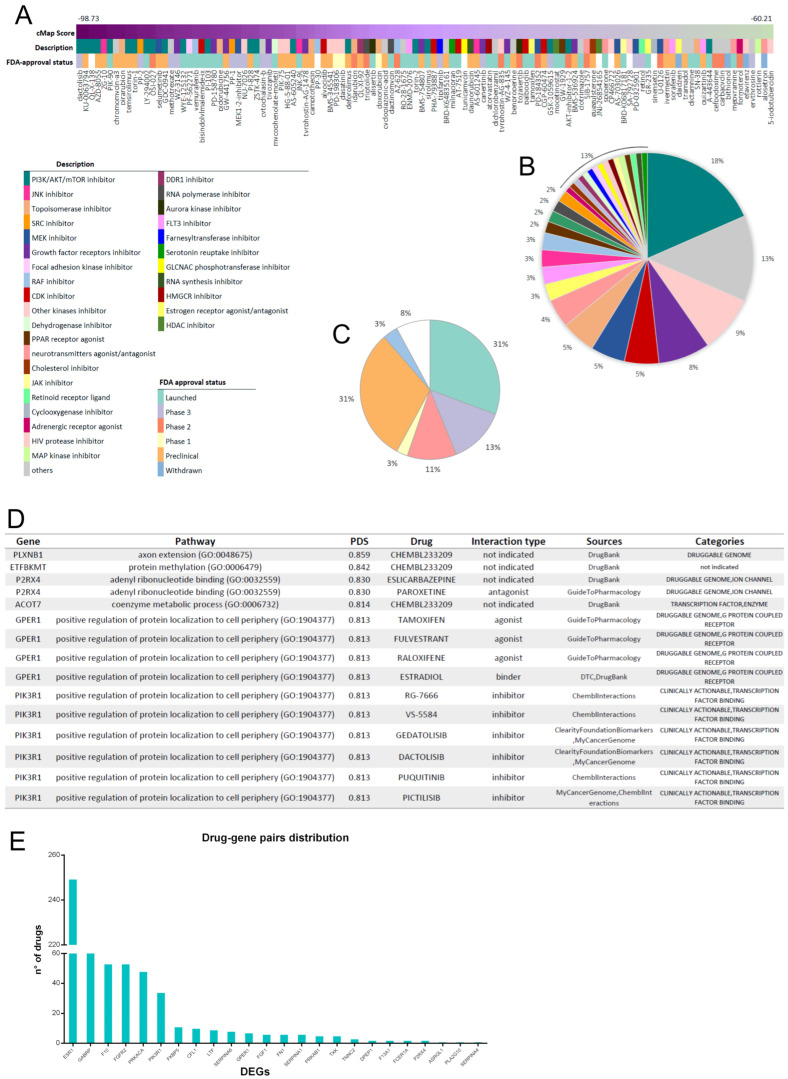
Drug repurposing: (**A**–**C**) Drugs identified through cMap querying, classified by CS, PCL, and FDA status. (**D**) Example of the dataset containing the list and all information relative to compounds identified through DGIdb querying. (**E**) Representation of the number of specific drugs found for each DEG in the DGIdb.

For the Signature-matching Approach, we used the dGES as input for the cMap which returned a list of perturbagens ranked by the Connectivity Score (CS). By applying the reversal method, we identified 115 compounds and six main PCL comprising most but not all the compounds (Figure 3A), potentially able to revert the metastatic phenotype. Most candidate drugs belong to mTOR, PI3K, SRC, MEK, Topoisomerase, and Growth factor receptors (such as IGF-1), inhibitor families, with a clear enrichment for molecules involved in interconnected pathways (MEK and PI3K/mTOR are parallel, and IGF-1 is one of their up-stream RTK). Other molecules that do not belong to the top-scoring PCL are also connected with the PI3K/AKT/mTOR pathway, for example, EGFR, RAF, CDK, and JNK inhibitors. Furthermore, in these interconnected families of drugs, it is interesting to note that some molecules, which are not part of the highly represented PCL, have a medium/high CS and are well-known drugs, used for a long time in clinical practice (methotrexate, mycophenolate-mofetil, dasatinib, tipifarnib, tamoxifen) (Figure 3B). Notably, one-third of the 115 compounds identified are launched, according to FDA approval status [46] (Figure 3C).

For the Pathway-based approach, we used Pathifier to establish the most altered pathways. From these pathways, we obtained two lists of differentially expressed genes (DEGs), 113 genes for GO_BP, and 78 genes from GO_MF. Then, we combined them to define a unique list of 121 most perturbed DEGs to be considered as potential pharmacological targets. They were used to query the DGIdb, which integrates about 15 pharmacological databases with the purpose to identify, for a gene of interest, drugs able to interact with its functions. We obtained a dataset comprising 575 compounds (Supplementary Table S2), for which Figure 3D provides a brief example. As expected, not all the input genes have a drug pair, while others are associated with several compounds (Figure 3E).

**Figure 4:**
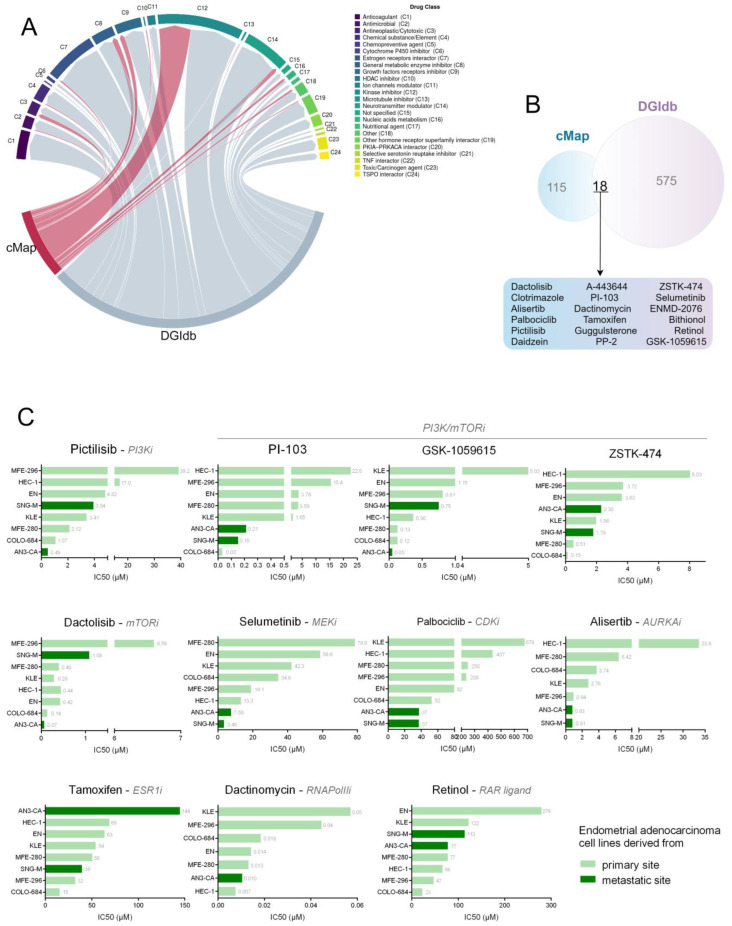
Validation of the efficacy of the identified drugs: (**A**) Chord plot summarizing the classification of the drug candidates obtained from the signature-matching and pathway-based repurposing approaches. Edge width is proportional to the number of drugs that belong to a certain drug class, highlighted by different colors and cited in the legend. (**B**) Venn diagram showing the molecules identified by both approaches. (**C**) Histograms showing IC50 values for each candidate drug found in the GDSC database, for EC cell lines. Next to each bar is the reported specific value of the IC50 for the corresponding cell line.

To get a robust prediction of drugs potentially able to restrain EC metastatization, we merged the results derived from cMap and DGIdb. Figure 4A, graphically represents how these compounds are distributed in 24 macro-categories related to their mechanism of action. The most represented are growth factors and hormone receptor inhibitors, estrogen receptor interactors, and kinase inhibitors. In particular, 18 compounds are common between the two lists (Figure 4B). Most of these drugs are inhibitors of the PI3K/AKT/mTOR pathway, cell cycle-related pathways (MEKs, CDKs, AURKs), or hormone/growth factor receptors (ESR1, RAR). Intriguingly, six compounds are launched (Dactinomycin, Palbociclib, Tamoxifen, Clotrimazole, GR-235, Retinol) and two of them are used for a specific disease, thus, suggesting that many of these drugs are yet defined as safe and effective, and are virtually ready to be used in clinical trials that specifically target metastatic EC.

### 3.4. In Silico Validation of the Efficacy of the Identified Drugs to Restrain Metastatic EC

To validate the ability of our predicted drugs to restrain EC metastatization, we took advantage of the GDSC database (24) and searched for the predicted compounds. Eleven out of 18 were found. For each of them, we queried GDSC to evaluate their effect on a list of endometrial cancer cell lines considering only endometrial adenocarcinoma to best mimic EC samples used for our analysis (Supplementary Table S3). Interestingly, five out of eleven compounds reduce the proliferation of metastatic EC cells more efficiently than EC cell lines derived from primary tumors. The other three drugs were effective even if the behavior of the metastatic cell lines was not consistent (Figure 4C). Above all, four of these drugs (Selumetinib, Alisertinib, Dactynomicyn, and Palbociclib) are launched or at least used for treating specific diseases, thus suggesting them as a possible ready-to-use treatment for metastatic EC.

## 4. Discussion

To our knowledge, this is the first time that drug repurposing is applied to EC by integrating different approaches.

We reasoned that reverting tumor phenotype to “normal” status is unlikely to be achieved in patients, but it would be more feasible to restrain tumor aggressiveness, by inhibiting its ability to metastasize, which is the deadliest feature of cancer cells. We identified a list of drug candidates using two drug repurposing approaches, one signature-matching and the other pathway-based, starting from transcriptomics data of EC patients. The combination of these two different computational approaches allowed us to exploit the strengths of both and minimize their weaknesses. Indeed, while the pathway-based method has a wider spectrum of candidate drugs, the signature-based method is more stringent. As a result, outputs overlap identified 18 drugs, pointing at these compounds as robust candidates to restrain EC metastatization and enhancing the reliability of our combined approach. In-silico validation highlighted eight of these compounds as very promising.

Pictilisib is the most promising among the identified PI3K/AKT/mTOR pathway inhibitors. It has been tested in several Phase 2 trials on different types of tumors both hematopoietic and solid, but it was never specifically used to treat endometrial or other gynecological cancers. To date, clinical trials with these PI3K/AKT/mTOR pathway inhibitors have shown few encouraging results, but still, high expectations and efforts are put into this class of drugs [2]. Indeed, the most recent recommendations of the European Society for Medical Oncology about EC, suggest focusing on predictive factors identification to select patients most likely to benefit from this type of therapy [26]. In this context, our findings suggest focusing on the comparison between M and NM EC patients for future PI3K/AKT/mTOR pathway inhibitors trials assuming that this patient stratification would unveil better responses which were not achieved so far.

Selumetinib, a MEK inhibitor, is registered in a total of 118 trials on different solid tumors, including one trial with EC patients (recurrent/resistant) [47]. The study results defined it as “tolerable”, even though it did not meet pre-trial specifications for clinical efficacy. It should be noted that it was used as a single-agent maintenance therapy in this trial. Theoretically, the combination of Selumetinib with other drugs and their use in not heavily treated patients could improve its efficiency. Moreover, it has been granted orphan drug status as a treatment for neurofibromatosis type 1 and as an adjuvant for thyroid cancer [48].

Alisertib is an AURKA inhibitor used in several clinical trials for many types of cancers, in particular hematopoietic tumors. Despite non-homogeneous results, its anti-tumor effect is compelling, and it remains under investigation both as monotherapy and in combination with chemotherapy [49,50].

Dactinomycin and Palbociclib are probably the most promising compounds since they are widely used in clinical practice. Dactinomycin is an RNA-polymerase inhibitor, thus a classical chemotherapy drug, but it has never been used for treating EC. Its extensive use in clinics ensures confidence in its scheduling and side effects management.

Palbociclib is a CDK inhibitor, approved for metastatic breast cancer (ER+, HER2−). Notably, among the 174 currently active trials, one is testing Palbociclib in combination with Abexinostat and Fulvestrant in gynecological tumors (ER+, HER2−) (NCT04498520). Another interesting trial is evaluating the combination of Palbociclib with Letrezole in metastatic-ER+ EC (NCT02730429). Of interest, this second trial almost mimics part of our results, since we observed that ESR1 expression is significantly increased in M compared to NM EC. Thus, we expect that the vast majority of metastatic EC are ER+ and we suppose that these tumors are the best candidates for our predicted drugs, including Palbociclib. The results of this trial will be of great interest for the clinical validation of our research, and it will enhance the reliability of our repurposing approach.

## 5. Conclusions

Our integrated approach enabled the identification of five drugs predicted to be highly efficient in reducing EC metastatization. Notably, they are already used in clinical practice which makes them excellent candidates to be tested as ready-to-use compounds to treat specifically metastatic EC. Moreover, our results suggest a new enrollment criterion for trials testing PI3K/AKT/mTOR pathway inhibitors, a class of drugs in which the medical community has high expectations, but that are still providing little results.

Collectively, these results serve as proof of principle demonstrating the robustness and reliability of our new, integrated repurposing approach. Further studies on larger and more balanced cohorts of samples will be needed to challenge this method which can be successfully applied not only to EC but also to other cancer settings.

## Data Availability

The datasets analyzed during the current study are openly available in the TCGA repository.

## Supplementary Materials

The following supporting information can be downloaded at: https://www.mdpi.com/article/10.3390/cells12050794/s1, Supplementary Table S1: Differential gene expression signature between metastatic (M) and non-metastatic (NM) EC; Supplementary Table S2: Dataset comprising 575 compounds obtained from Pathifier analysis; Supplementary Table S3: Endometrial adenocarcinoma cell lines from GDSC.
